# RETRACTION: Effect of Drainage Versus No Drainage after Thyroid Surgery on Wound Complications, a Meta‐Analysis

**DOI:** 10.1111/iwj.70377

**Published:** 2025-03-21

## Abstract

**RETRACTION:** ZhangL. , WuY. , LiuX. , HanJ. , and ZhaoJ. , “Effect of Drainage Versus No Drainage after Thyroid Surgery on Wound Complications, a Meta‐Analysis,” International Wound Journal 20, no. 10 (2024): 4023–4030, 10.1111/iwj.14291.PMC1068153037400984

The above article, published online on 03 July 2023, in Wiley Online Library (http://onlinelibrary.wiley.com/), has been retracted by agreement between the journal Editor in Chief, Professor Keith Harding; and John Wiley & Sons Ltd. Following an investigation by the publisher, all parties have concluded that this article was accepted solely on the basis of a compromised peer review process. The editors have therefore decided to retract the article. The authors did not respond to our notice regarding the retraction.

**RETRACTION:** L. Zhang , Y. Wu , X. Liu , J. Han , and J. Zhao , “Effect of Drainage Versus No Drainage after Thyroid Surgery on Wound Complications, a Meta‐Analysis,” International Wound Journal 20, no. 10 (2024): 4023–4030, 10.1111/iwj.14291.PMC1068153037400984

The above article, published online on 03 July 2023, in Wiley Online Library (http://onlinelibrary.wiley.com/), has been retracted by agreement between the journal Editor in Chief, Professor Keith Harding; and John Wiley & Sons Ltd. Following an investigation by the publisher, all parties have concluded that this article was accepted solely on the basis of a compromised peer review process. The editors have therefore decided to retract the article. The authors did not respond to our notice regarding the retraction.

